# Features of Selective Sorption of Neodymium and Praseodymium Ions by Interpolymer Systems Based on Industrial Sorbents KU-2-8 and AV-17-8

**DOI:** 10.3390/polym17040440

**Published:** 2025-02-07

**Authors:** Jumadilov Talkybek, Kamil Kabzhalelov, Zamira Malimbayeva, Zhanar Korganbayeva

**Affiliations:** 1 Laboratory of Synthesis and Physicochemistry of Polymers, JSC Institute of Chemical Sciences after A.B. Bekturov, Sh. Valikhanov St. 106, Almaty 050010, Kazakhstan; kamil_kabhzalelov@outlook.com (K.K.); malimbayeva.zamira@gmail.com (Z.M.); 2 Institute of Natural Sciences and Geography, Abai Kazakh National Pedagogical University, Dostyk Ave. 13, Almaty 050010, Kazakhstan; korganbaeva.zhan@mail.ru

**Keywords:** interpolymer system, neodymium ions, praseodymium ions, selective extraction, ion exchangers AB-17-8 (Cl^−^), KU-2-8 (Na^+^), remote interaction

## Abstract

In this study, the possibilities of selective sorption of neodymium and praseodymium ions from a mixture of their solution using interpolymer systems composed of two industrial sorbents, cation exchangers KU-2-8 (Na^+^) and AB-17-8 (Cl^−^), at different molar ratios were investigated. The processes of sorption and desorption were carried out in two modes: dynamic (with active stirring) and static (without stirring the working solution from which REEs were extracted). According to the obtained results, sorption in the dynamic mode in the ratios of 4:2 and 3:3 does not lead to any selectivity. However, a high degree of extraction was noted for both ions: Pr^3+^ = 99.36%, Nd^3+^ = 95.67% for the 4:2 system and Pr^3+^ = 81.33%, Nd^3+^ = 79% for the 3:3 system. In the static mode, the degree of extraction of both metals was significantly lower: for the 4:2 system Pr^3+^ = 19.33%, Nd^3+^ = 24%, but greater selectivity with respect to neodymium was observed. With a ratio of 4:2, neodymium was sorbed better than praseodymium by 24.16%, and in the 3:3 system by 39.83%. When desorption from the cationite was carried out with nitric acid, a similar tendency was preserved. Thus, it was shown that interpolymer systems can be used in industry by varying the conditions of sorption and desorption for the successful extraction of neodymium and praseodymium from industrial solutions.

## 1. Introduction

Currently, there is growing industrial demand for rare earth elements (REEs), as these elements are now an integral part of the production of many carbon-neutral technologies. Interest has especially increased in secondary sources due to the huge potential for their recycling [1]. An important difficulty is the separation of rare earth metals from each other. The main methods for extracting REEs from both primary and secondary sources are precipitation, extraction, and sorption [2,3,4,5,6]. The importance of the efficiency of the technological process for obtaining REEs (rare earth metals) is explained by the fact that in recent years, the demand for REEs has grown rapidly, as they are used in high-tech and environmentally friendly technologies, such as high-performance magnets, rechargeable batteries, and low-energy fluorescent lamps [7]. Praseodymium is used to produce magnets, optical fibers, and carbon-arc lamps; for petroleum refining and automotive catalysts; and as an additive. Neodymium is used to produce magnets incorporated into electronics, such as computers and cell phones; also, this element is the basic for NdFeB magnets [8,9]. Therefore, the aim of this study was to determine the conditions of selectivity of intergel systems based on KU-2-8 (Na^+^) and AB-17-8 (Cl^−^) in relation to praseodymium and neodymium ions (metals that are relevant in many industries and high technologies) in static and dynamic sorption and desorption modes. This goal was achieved by completing the following tasks: (1) to determine the dynamics of sorption of each ion individually from a model solution with a concentration of 100 mg/L by interpolymer systems composed of a cation exchanger and anion exchanger in different ratios; (2) to select the ratios at which maximum sorption occurs based on the obtained data; (3) to use the selected systems to study sorption of neodymium and praseodymium from a mixture of their solution in different modes; (4) to determine whether there is selectivity for one of the ions for the selected systems to solve the urgent problem of separating REEs from each other. We used these ion exchange resins because they are cheap, accessible, and because sorption using such systems is an environmentally friendly [10] method aimed at the selectivity and concentration of rare earth metals from solutions. We assume that with the help of the “long-range effect”, KU-2-8 and AV-17-8 additionally swell and change their conformations. This in turn can contribute to the following: firstly, a general improvement in sorption indicators (such as the degree of extraction), and secondly, the appearance of selectivity to one of the ions. The novelty of this work lies in (1) demonstrating that the sorption process using interpolymer systems in a static mode differs from the dynamic one; (2) at different ratios of cation exchanger to anion exchanger, different selectivity is observed in relation to the studied metal ions.

The relevance of such searches is confirmed by a huge number of works devoted to the possibilities of extracting REEs from solutions obtained by leaching ores, as well as from processed raw materials. Thus, in a review study [11], the authors confirm that sorption processes of all possible methods are the most relevant and most suitable for the environment. The article lists such modern developments as polymers with ion imprints, porous organic polymers, chelate ion exchangers, silicate adsorbents (based on covalently and non-covalently bound ligands), metal–organic frameworks, magnetic adsorbents, as well as modified materials from nanocarbon.

However, the extraction and separation of REEs remain expensive and complex, creating numerous environmental problems [12]. Recently, solvent extraction has become a popular method for recovering REEs from liquid wastes, but its dependence on flammable and toxic organic solvents makes it unsuitable for the treatment of industrial wastewater with low concentrations of rare earth elements. In contrast, adsorption, which binds metal ions to an adsorbent, is more environmentally friendly, reduces the use of solvents and additives, allows for a wider range of concentrations, and is similar in efficiency. Given the stringent environmental regulations, adsorption is commercially superior to solvent extraction [13]. A variety of adsorbents have been investigated for the recovery of REEs from ores and municipal solid wastes, including activated carbon, carbon nanotubes, dried biomass, nanocomposites, polymeric materials, silica, and zeolites [14]. The adsorption of REEs is influenced by various mechanisms, each of which is driven by specific intermolecular forces. A special role is played by the mechanisms of chemical coordination between the metal ion and the adsorbent.

A previous study [15] devoted to analog materials based on porous sorbents also indicates the advantages of adsorption compared to other methods of extracting target metals. The development of effective porous organic polymers based on carbon, porous silicon, and porous metal–organic frameworks is the leading direction today. But the main problem with such materials is the complexity of their manufacture and, therefore, their high cost.

A possible solution to the problems of high cost and complexity of manufacturing highly selective adsorbents may be the use of conventional sparsely cross-linked industrial hydrogels based on the “long-range effect” or, in other words, the “remote interaction effect”. The use of cross-linked polyelectrolytes is also justified from an environmental point of view. In this case, there is no need to use toxic and flammable extractants, sorption can be carried out in aqueous solutions, and traditional cationites and anionites can be used repeatedly. The effect of this effect was studied in [16,17,18,19,20,21] and is largely associated with the behavior of macromolecule charges. Polyelectrolyte molecules dissociate in solution, which leads to the appearance of macroions and low-molecular ions. Accordingly, if the solution contains cationites and anionites that have opposite charges of macroions and counterions, this leads to mutual activation of ionites. Counterions dissociate more effectively in solution, thereby stretching the macromolecule, changing its conformation and leading to greater ionization and swelling.

The phenomenon of remote interaction largely affects the physicochemical properties of hydrogels (both cationites and anionites). The effect can be observed by changing such characteristics of hydrogels in intergel systems of various ratios as the pH of the solution, electrical conductivity, degree of swelling, and the amount of sorption of rare earth metal ions. Thus, in [22,23], the features of sorption of rare earth metals by various polyelectrolyte systems and changes in the properties of cationites and anionites during mutual activation were studied. In [22], Amberlite IR120 and AB-17-8 ion exchangers were used for the sorption of europium ions. It was found that the molar ratios of Amberlite IR120:AB-17-8 = 4:2 and 1:5, due to the effect of remote interaction, provide a significant increase in the following sorption properties: the degree of sorption, degree of polymer chain binding, and effective dynamic exchange capacity. In [23], KU-2-8 and AV-17-8 were used to study the dynamics of sorption of cerium ions. In this study, it was found that, compared to individual the cation exchanger and anion exchanger, the system in the ratio of 3:3 (KU-2-8:AV-17-8) showed higher rates of cerium extraction from the solution. Also, in this system, an increase in electrical conductivity was found due to greater ionization of two sorbents located in one solution.

## 2. Materials and Methods

### 2.1. Equipment

The mass of the sorbents was determined by weighing on a Shimadzu TX423L electronic analytical balance. The residual concentration and concentrations after desorption were determined using a KFK-3 photocolorimeter and atomic emission analysis (Thermo Scientific™ iCAP™ PRO XP ICP-OES, Thermo Fisher Scientific Inc., Waltham, MA, USA). The measurement error was less than 1%. For the measurement of the hydrogen ion concentrations in the solutions, a “Metrohm 827 pH–Lab” pH meter (Metrohm AG, Herisau, Switzerland) was applied. FTIR spectra of the initial ion-exchange structures and the interpolymer system was obtained with using a NICOLET 5700 spectrophotometer (Thermo Fischer scientific, Waltham, MA, USA). TGA (Model: SKZ1053, SKZ International Co., Ltd., Jinan, Shandong, China) was used for the determination of the thermogravimetric properties of the initial ion-exchange structures and the interpolymer system. TGA/DSC conditions: dynamic mode; interval of temperature—20–350 °C (for KU-2-8), 20–400 °C (for AV-17-8); heating rate 10 °C/min; air atmosphere.

### 2.2. Materials

**Figure 1:**
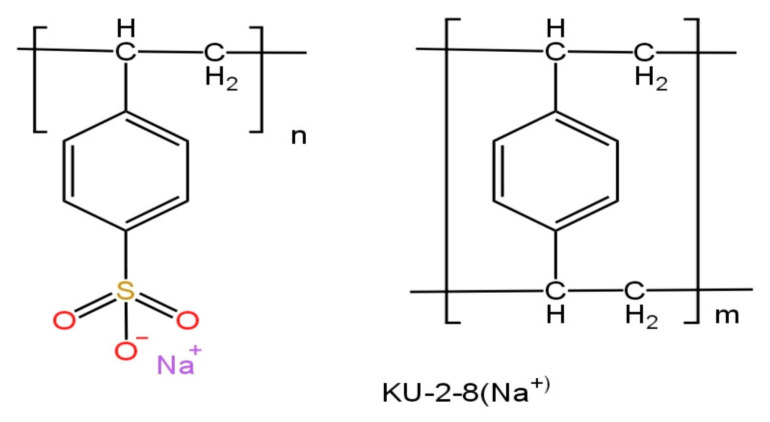
The chemical formula of KU-2-8 ion exchanger (salt form).

**Figure 2:**
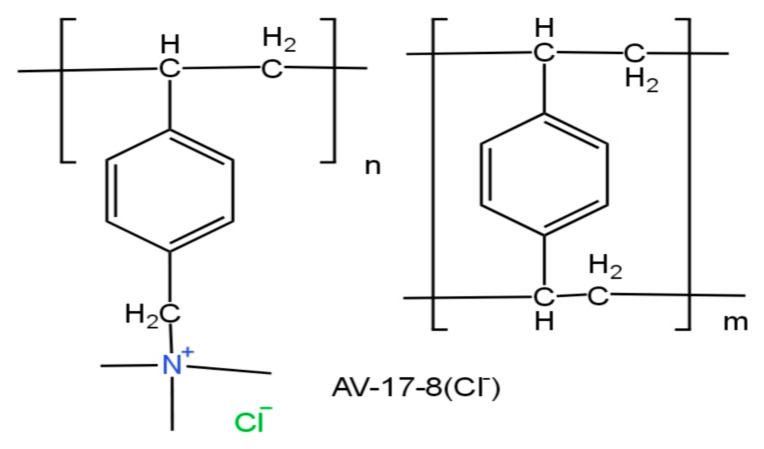
The chemical formula of AV-17-8 ion exchanger (salt form).

The studies were conducted in solutions of hexahydrate neodymium and praseodymium nitrate (both salts; Sigma-Aldrich, Darmstadt, Germany). Concentrations of Nd^3+^ and Pr^3+^ = 30 mg/L each in the mixture and concentrations of Nd^3+^ and Pr^3+^ = 100 mg/L were used when studying the sorption dynamics individually for each ion. Ion-exchange resins in salt forms: KU2-8 (Na^+^) strongly acid (Azot, Cherkassy, Ukraine) cation exchanger and strongly basic AV-17-8 (Cl^−^) (Azot, Cherkassy, Ukraine) gel-type anion exchanger based on a copolymer of styrene and divinylbenzene were used in a dried state to study the sorption of target ions. Figure 1 and Figure 2 show simplified structures of the cation exchanger and anion exchanger. Table 1 presents some characteristics of the ion exchange resins that were used in this study. To conduct this study, interpolymer systems with different molar ratios of cation exchanger and anion exchanger were composed from these hydrogels. Nitric acid (Sigma-Aldrich) was used for desorption.

### 2.3. Experiment

The experiments were carried out at room temperature. The moisture content in KU-2-8 (Na^+^) was 17%, and in AV-17-8 (Cl^−^) 32.34%. The studies of the interpolymer system were carried out in the following order: each ion-exchange resin was placed in dry form in separate polypropylene grids, in different molar ratios KU-2-8:AV-17-8 (from 6:0—the initial cation exchanger, with mass 0.12 g; 5:1 (0.1 g:0.021 g); 4:2 (0.079 g: 0.041 g); 3:3 (0.059 g: 0.062 g); 2:4 (0.039 g:0.082 g); 1:5 (0.019 g: 0.10 g); and 0:6 (0.123 g)—only anion exchanger. Then, the polypropylene grids with ion-exchange resins KU2-8 and AV-17-8 were placed in glasses with solutions of neodymium and praseodymium nitrate (200 mL each), when studying the dynamics of sorption of each metal separately. Exactly the same procedure was performed for a solution of a mixture of these ions. Separate experiments with each REE were conducted in a static mode (without stirring), and aliquots were collected after 1, 6, 24, and 48 h from the start of the sorption process. The residual concentration during sorption of each ion individually was determined using a photocolorimeter according to the method in [27]. In the case of the experiment with a mixture of metals, both modes were used, static and dynamic (with active stirring, speed range 80–150 rpm), and aliquots for atomic emission analysis were collected after 48 h of sorption. In this study, the pH level of the solution was not adjusted by adding acids or alkalis. This decision was due to the fact that setting a certain pH level could affect or neutralize the “long-range effect” between the cationite and the anionite. Thus, the pH value in the solution from which sorption was carried out depended only on the amounts of sorbents in the system. Based on the data obtained on the dynamics of sorption of each REE separately, the cation exchanger and anion exchanger molar ratios were selected: 4:2 and 3:3. After sorption, the polypropylene grids with sorbents were removed from the initial solution and placed in glasses with 2% nitric acid (200 mL) to carry out desorption. Desorption was also carried out in two modes for 72 h. After this period, aliquots were collected. The main objective was to test the presence of selectivity towards low molecular weight ions in these interpolymer systems.

## 3. Results and Discussion

### 3.1. Study of Sorption of Target Metal Ions by Interpolymer Systems

**Figure 3:**
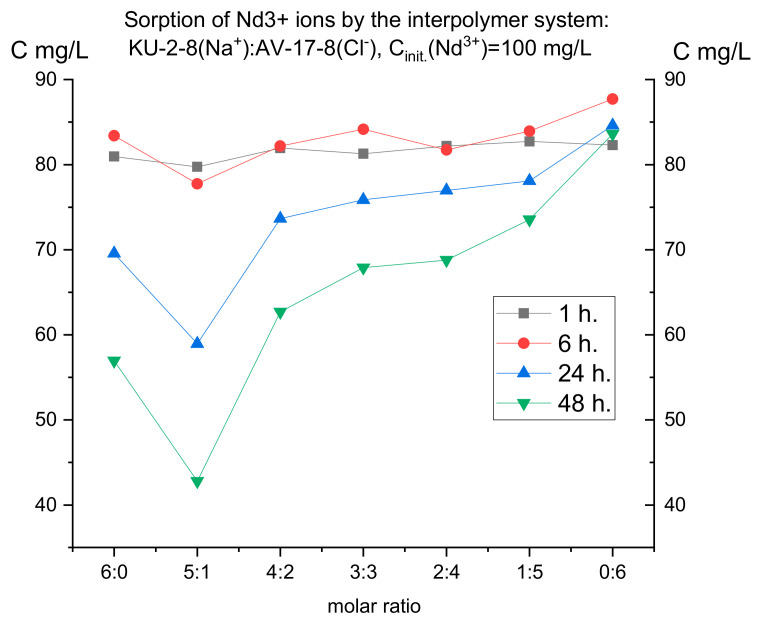
Residual concentration of neodymium ions at different molar ratios of hydrogels over time.

**Figure 4:**
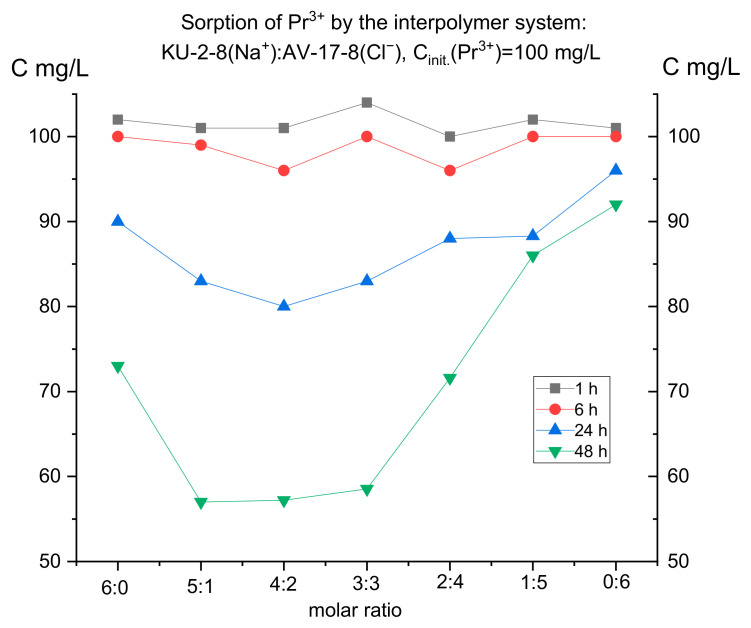
Residual concentration of praseodymium ions at different molar ratios of hydrogels over time.

For selective extraction of one of the presented metals, it was necessary to initially establish the features of their individual sorption. For this purpose, the dynamics of absorption were studied separately for 48 h using model solutions with a concentration of 100 mg/L of each metal (neodymium and praseodymium) (taking aliquots after 1, 6, 24, and 48 h, respectively). Figure 3 and Figure 4 present the results of ion sorption at different molar ratios of hydrogels (cation-exchange resin and anion-exchange resin).

It is interesting to note that, despite the close ionic radius, oxidation state, and mass, the curves on the graphs of these metals differ. This means that each ion has its own characteristic absorption dynamics by the interpolymer systems KU-2-8 (Na^+^) and AB-17-8 (Cl^−^), which can presumably be used for the selective extraction of one metal in several cycles. It is also noticeable on both graphs that, compared to an individual cationite (6:0), systems composed of cationite and anionite pairs show higher sorption efficiency. This is observed despite the fact that in the 6:0 ratio, the cationite mass is the largest and it is KU-2-8 that sorbs the target ions. In Figure 3, the lowest residual metal concentration is observed in the 5:1 system, although in this ratio, the cationite mass is less than in the 6:0 ratio. If the anionite did not show any effect, we would observe a dependence close to linear in the increase in the residual metal ion concentration in the solution with a decrease in the KU-2-8 mass (from 6:0 to 1:5). In Figure 3 and Figure 4, after 48 h of interaction, we do not observe anything resembling a linear dependence. This confirms the long-range effect between the cationite and the anionite, by means of which mutual activation of KU-2-8 and AB-17-8 occurs, which leads to greater ionization of the two polyelectrolytes. In Figure 4, we observe that the 5:1, 4:2, and 3:3 systems show the lowest residual concentration of praseodymium after 48 h of interaction, surpassing the individual cationite (6:0).

**Figure 5:**
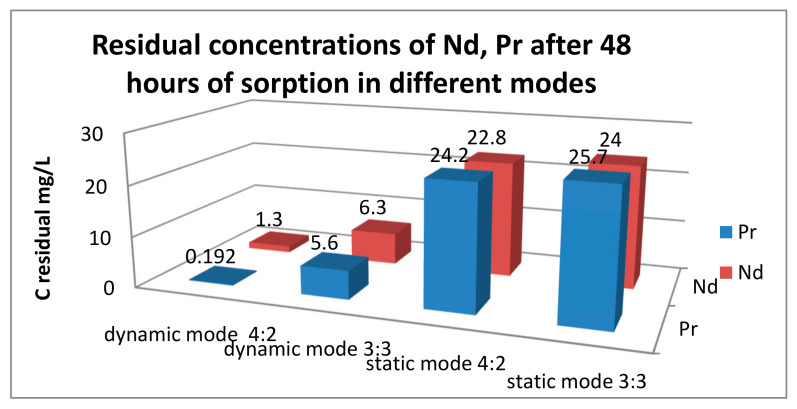
Residual concentrations of neodymium and praseodymium ions after 48 h of sorption.

Thus, for praseodymium, after 48 h of sorption, the lowest residual concentrations are observed at PE (polyelectrolyte) ratios of 5:1, 4:2, and 3:3. For neodymium, after the same amount of time, the highest absorption is observed mainly only in the ratio of 5:1. Therefore, for further study of the issue of possible selective sorption using these results for each ion separately, interpolymer systems can be selected. Based on the fact that in the case of neodymium there were no peaks of increased sorption in the ratio of 4:2 and 3:3, unlike praseodymium, it is logical to select these molar ratios of hydrogels and not take into account the other systems. Thus, we make an assumption that it is in these systems that we can obtain selectivity with respect to praseodymium ions. To test this idea, cation exchanger and anion exchanger molar ratios of 4:2 and 3:3 were used in a mixture of neodymium and praseodymium solutions. The initial concentration of each ion was 30 mg/L. In this case, sorption was carried out in two modes: dynamic (with active mixing) and static (without mixing the model solution of the metal mixture). This solution was aimed at testing the possibility of influencing the choice of mode on the selectivity of extraction (since in the case of active mixing, equilibrium is established faster, and even if one of the ions binds to the polymer matrix more energetically favorably, the kinetic factor can negate this small difference). Table 2 presents the results of the residual concentration of the target metals after 48 h of sorption. As can be seen from the numerical values, with constant mixing, the residual concentration of both metals is significantly lower than in the static mode. Also of interest is that there is better sorption of Pr^3+^ ions in the dynamic mode, and vice versa, better sorption of Nd^3+^ in the static mode. This partly contradicts our expectations, since in the case of individual sorption in the ratios of 4:2 and 3:3 in static mode, we expected higher absorption of praseodymium. Therefore, in a static mode, based on the study of the sorption characteristics of each ion separately from model solutions, it is not always possible to accurately predict how a given ion will be sorbed in a mixture with another metal. Figure 5 shows the final graph for the residual concentrations of the ions under study.

Adsorption capacity (Table 3) is a quantitative characteristic of the sorption material, showing the amount of substance (adsorbate) that can be absorbed by a unit of mass or volume of the adsorbent. This value was calculated using the following formula:

$$q=\frac{{(C}_{0}-C_{f})*V}{m}$$

where *q* (mg/g) is the adsorption capacity, *C*_0_ is the initial concentration, *C_f_* is the final concentration, *V* is the volume of the solution, and *m* is the mass of the sorbent.

The literature provides data on the sorption capacity of various sorbents in relation to rare earth metals. For example, in works on sorption for neodymium, this indicator was functionalized lignin-activated carbon (q = 335.5 mg/g) [28], commercial activated carbon (CAC) 19.1 mg/g [29], and sawdust biochar 8 mg/g [30]. For praseodymium, this indicator was determined for various sorbents: maximum sorption capacity of Arthrospira platensis biomass—99.3 mg/g [31]; clinoptilolite under optimal conditions—47.5 mg/g [32]; hydrogenated Dowex 50WX8 resin—30 mg/g [33]. The results regarding sorption capacity show the high potential of the sorbents used in this work for the extraction of target metals.

As stated above, in this study, we did not set a specific pH level by adding acids or alkalis. This decision was made based on the assumption that a given pH level can neutralize the long-range effect between the cation exchanger and the anion exchanger placed in the same solution. Therefore, the pH depended only on the ratios of the polyelectrolytes in the solution and their long-range interaction with each other. The results of measuring the pH with constant stirring and in static mode at different time intervals are presented in Table 4.

To answer the most important question about the possibility of selectivity, it is necessary to calculate the extraction rates of praseodymium and neodymium and to calculate per 1 mole of polymer, assuming that the anionite does not participate in sorption. This approach allows for eliminating the influence of differences in the mass of sorbents at different ratios of KU-2-8:AV-17-8 and objectively assessing the efficiency of sorption of each metal. It is obvious that due to the presence of a greater mass of cationite in the ratio of 4:2 leading to better sorption, we may be prevented from seeing the overall picture. In the initial experiment, we studied the sorption of each ion separately, we considered all the ratios of KU-2-8:AB17-8 from 6:0, where only the cationite was present, to 0:6, where only the anionite was present. The minimum mass of the cationite was in the ratio of 1:5 equal to 0.19 g; the maximum mass of KU-2-8 was in the 6:0 system (0.12 g), which is almost six times greater than the ratio of 1:5. Therefore, the conversion to 1 mole consists of reducing to the minimum mass of the cationite and how effectively (the degree of extraction per 1 mole) this minimum mass of the sorbent is able to extract each of the metals from the solution of their mixture. Table 5 presents the results of the extraction rate for each ion. At first glance, it is clear that the greatest difference in the percentage of extraction rate is observed in the 3:3 static mode.

The degree of extraction (sorption) of both ions was calculated using the following formula:

$$\eta =\frac{C_{init.}-C_{resid.}}{C_{init.}}*100\%$$

where *C_init._*—initial concentration of neodymium ions in solution, mg/L; *C_resid_*_._—residual concentration of neodymium ions in solution, mg/L.

The degree of extraction per 1 mole is calculated using the following formula:

$$\epsilon =\frac{\eta }{n}$$

where *ϵ* is the degree of extraction per 1 mole, *η* is the degree of extraction, and *n* is an integer showing how much the given mass of cationite is greater than the minimum in a ratio of 1:5.

In the static mode, the degree of extraction of both metals was significantly lower than in the dynamic mode: for the 4:2 system, Pr^3+^ = 19.33%, Nd^3+^ = 24%. But, despite this, in the 4:2 system, neodymium was sorbed better than praseodymium by 24.16%, and in the 3:3 system by 39.83%. Sorption in the dynamic mode in the ratios 4:2 and 3:3 does not lead to any selectivity (praseodymium ions were sorbed better than neodymium ions by 3.93% and 2.96% for each ratio). However, a high degree of extraction of both ions was noted: Pr^3+^ = 99.36%, Nd^3+^ = 95.67% for the 4:2 system and Pr^3+^ = 81.33%, Nd^3+^ = 79% for the 3:3 system. In general, a higher difference in the values of the degree of extraction in the static mode is clearly visible, which may indicate that despite the rapid establishment of equilibrium with active mixing, it may be more advantageous to carry out the sorption process in a stagnant solution to obtain greater selectivity. In this case, a contradiction arises: the choice of a dynamic mode to accelerate the sorption process and a greater degree of extraction of each metal, or the choice of a static mode with lower indicators of the degree of extraction and a long establishment of equilibrium, but slightly greater selectivity with respect to one of the target ions.

Two other important sorption indices, the total degree of polymer chain binding and the effective dynamic sorption capacity, were calculated to provide a complete picture of the sorption characteristics of each target ion under different interaction conditions. Those results are shown in the Table 6 and Table 7.

The total degree of binding of the polymer chain refers to a quantitative measure of how many binding sites or functional groups within a polymer chain are engaged in interactions with other molecules, ions, or surfaces. The total degree of binding of the polymer chain was determined by the following formula [22]:

$$\theta =\frac{\nu_{sorb.}}{\nu }*100\%$$

where *ν_sorb._* is the amount of sorbed metal, mol; *ν* is the amount of polymer sample, mol.; θ—total degree of binding of the polymer chain.

Effective dynamic sorption capacity is a quantitative characteristic of a sorption material that determines the amount of substance that the material can effectively retain in a dynamic mode. The effective dynamic sorption capacity was calculated using the following formula, where we changed the numerator from the mass of the sorbed metal to its amount of substance [22]:

$$Q=\frac{\nu_{sorb.}}{m_{sorbent}}$$

where *ν_sorb._* is the amount of sorbed metal, mol; *m_sorbent_* is the mass of the sorbent.

Here, we can also observe large differences between the different sorption modes. With constant stirring, the degree of polymer chain binding is four times higher than the same indicator for sorption in a stagnant solution. The same results can be observed for the effective dynamic sorption capacity. In dynamic mode, this indicator is 4–5 times higher than in static mode. There was a tendency for praseodymium to be better absorbed in dynamics, and neodymium in static mode was preserved for the two above-mentioned indicators.

### 3.2. FTIR Spectra of the Initial Ion Exchangers and the Interpolymer Systems

**Figure 6:**
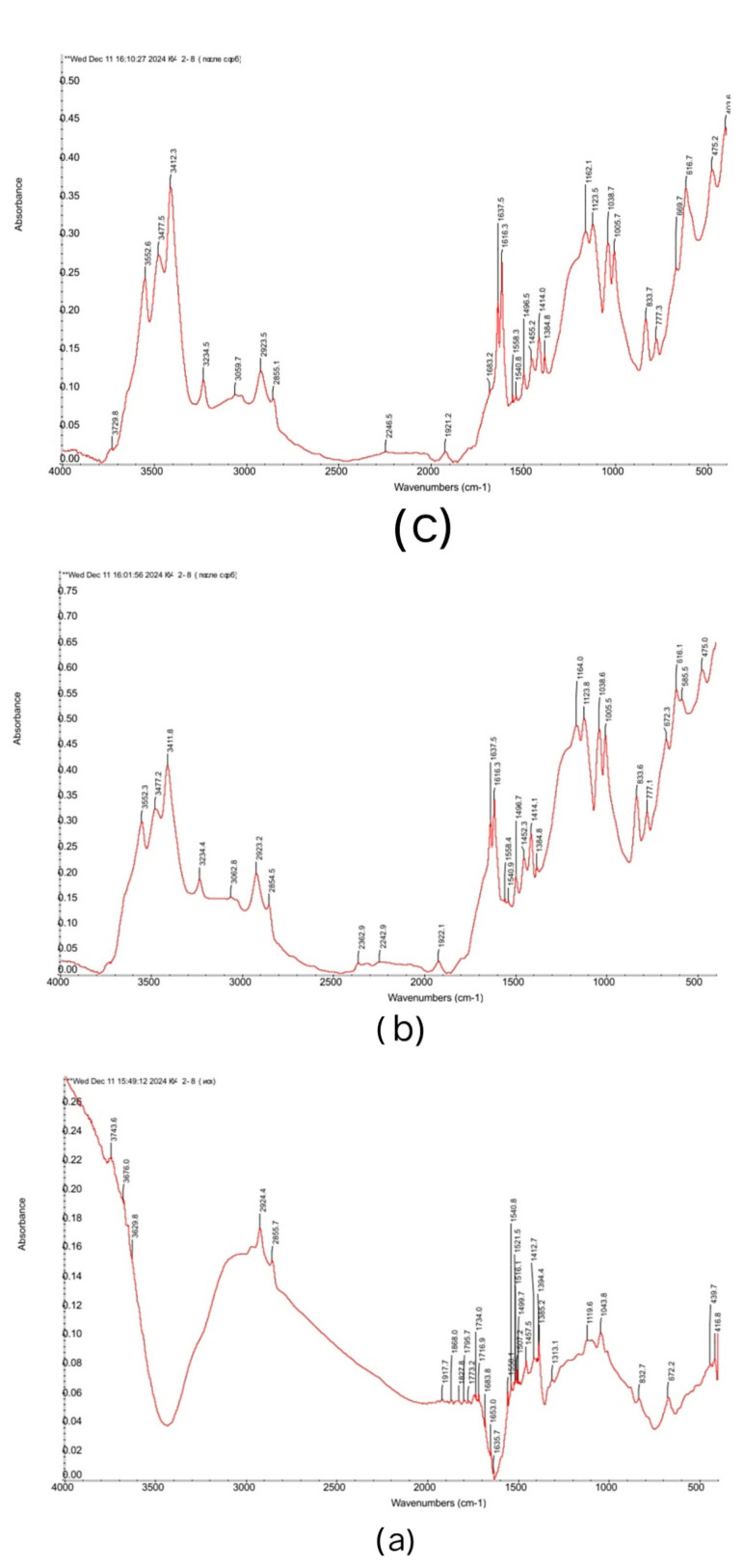
Fourier transform infrared (FTIR) spectra of (**a**) KU-2-8 (Na^+^) initial; (**b**) KU-2-8 4:2 after sorption; (**c**) KU-2-8 3:3 after sorption.

Figure 6 shows the IR spectra of the initial KU-2-8 (without sorbed neodymium and praseodymium), KU-2-8 from the 4:2 system, and KU-2-8 from the interpolymer pair in a 3:3 ratio 48 h after the start of the interaction.

By analyzing these IR spectra, one can see the characteristic absorption bands of the –SO_3_^−^ functional group and, in general, for the cationite KU-2-8. These include the absorption bands (a)—initial KU-2-8: 672.2 (C-S bound) [34]; region 1200–1000 corresponding to –SO_3_^−^ group [35] (1043.8, 1119.6). In KU-2-8 (b), (4:2 after sorption): 672.3 (C-S); 1164.0, 1123.8, 1038.6, 1005.5 (–SO_3_^−^ group). In KU-2-8 (c), (3:3 after sorption): 669.7 (C-S); 1162.1, 1123.5, 1038.7, 1005.7 (–SO_3_^−^ group). Bands in the IR spectrum in the region of 2925–2923 and 2856–2854 are characteristic of C-H assym. and sym. str. of CH_2_ [36]. Bands between 1616 and 1400 mainly show characteristic peaks for benzene ring and CH groups. The absorption bands in the 3700–3200 region are caused by the possible presence of moisture, so this region is of little information. This is also confirmed by the absorption in the 1637–1635 area (OH deform. of H_2_O). The overall picture when comparing the IR spectra of the initial cation exchanger with the samples after sorption shows obvious changes due to the absorption of target metals into the polymer matrix. Samples b and c are almost completely identical to each other in IR spectrum and differ from the original KU-2-8 (a). Also, in samples (b) and (c) after sorption, new peaks appeared at 616.1, 585.5, and 475.0, and 616.7 and 475.2, respectively. So far, we cannot say for sure whether these absorption areas have any new information, they may indicate the appearance of coordination bonds between metal ions and oxygen in the functional group of the cation exchanger. But ultimately, we can say with confidence that sample (a) (cationite before sorption) differs from samples (b) and (c), which confirms that the sorption process has occurred.

**Figure 7:**
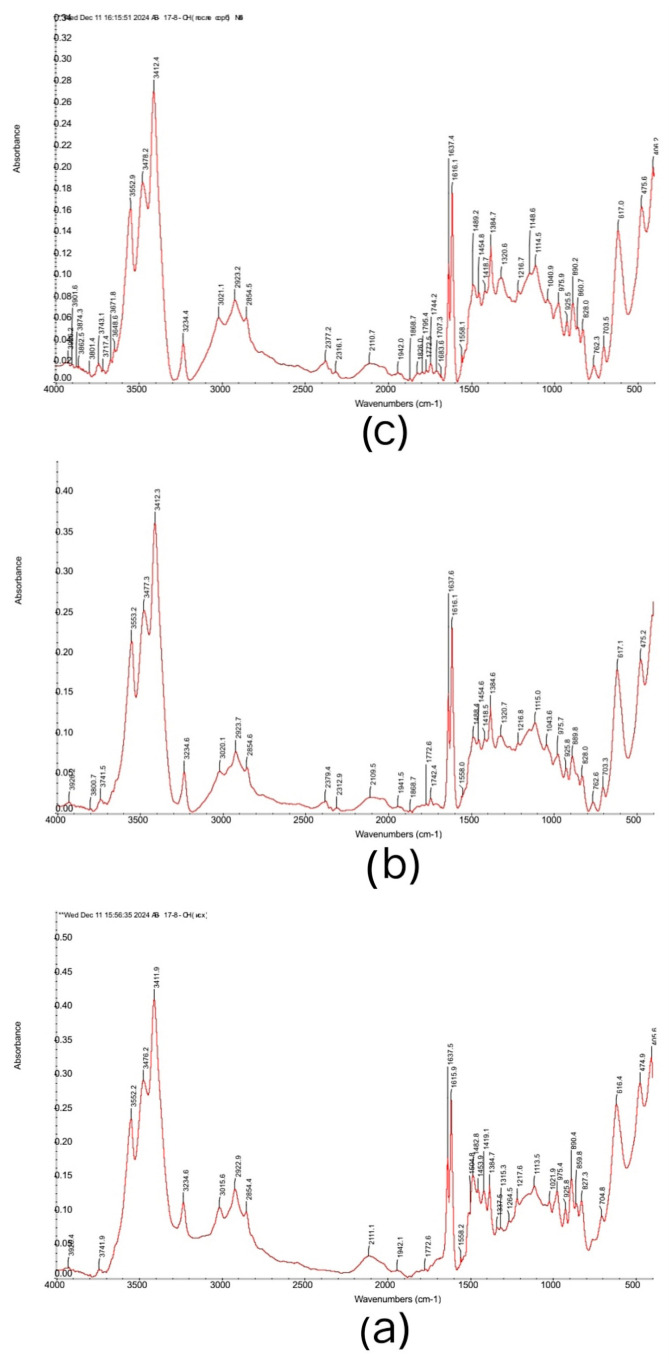
Fourier transform infrared (FTIR) spectra of (**a**) AV-17-8 (Cl^−^) initial; (**b**) AV-17-8 4:2 after sorption; (**c**) AV-17-8 3:3 after sorption.

Figure 7 presents FTIR spectra of initial the AB-17-8 (without sorbed ions), AB-17-8 from the 4:2 system, and AB-17-8 from ratio 3:3 from time of interaction 24 h.

The IR spectrum of three samples of AB-17-8 also shows an interesting picture, where (a) is the initial anionite, (b) is the anionite from the ratio 4:2, and (c) is the anionite from the ratio 3:3 after sorption. From this figure, it is evident that the spectrum of the initial anionite is almost identical to the spectrum of AV-17-8 after sorption. This indicates that there were no significant changes in the structure of AV-17-8, due to the fact that this polymer did not sorb neodymium and praseodymium cations. This is consistent with the simple logic that the anionite will not absorb cations from the solution. Therefore, in interpolymer systems, the role of the anionite is precisely in mutual activation together with the cationite, which leads to greater ionization of the two sorbents, to greater swelling, and, accordingly, to improved sorption indices. Characteristic bands in the spectrum for the tertiary ammonium functional group: in initial (a) AV-17-8 (Cl^−^): 925.8, 890.4, 859.8, 827.3 (-N^+^ (CH_3_)_3_), 1482.8 (C-N vibration) [36,37]; (b) AV-17-8 (4:2 system): 925.8, 889.8, 828.0 (-N^+^ (CH_3_)_3_), 1488.4 (C-N vibration); (c) AV-17-8 (3:3 system): 925.5, 890.2, 860.7, 828.0 (-N^+^ (CH_3_)_3_), 1489.2 (C-N vibration). Also, the area 3700–3100 and peak 1637 indicate the presence of moisture in three anionites. The peak around 2923 describes CH assym. of CH_2_ and CH_3_ in CH_3_-N. The peak around 2854 indicates sym. C-H str. of CH_2_ and CH_3_ in CH_3_-N [29]. The appearance of a weak peak of 762.6 for AV-17-8 in the 4:2 system and 762.3 for AV-17-8 in the (3:3) system and bands 1772.6, 1772.5 [38] may indicate the sorption of nitrate anions that were part of the rare earth metal salts. To summarize, using IR spectra, we confirmed that the anionite does not undergo major changes after the sorption process compared to the cationite KU-2-8.

### 3.3. TGA Analysis of the Initial Ion-Exchangers and the Interpolymer Systems (4:2 and 3:3)

**Figure 8:**
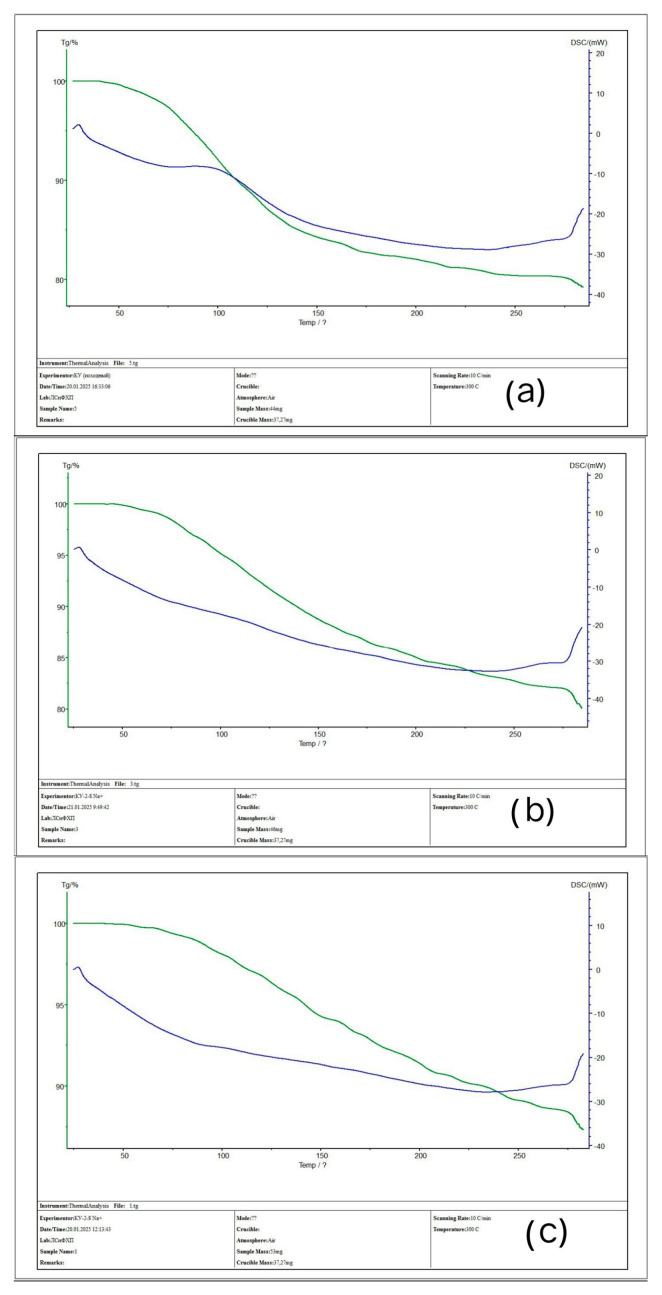
TG (green curve) and DSC (blue curve) graphs: (**a**) initial KU-2-8 (not involved in sorption); (**b**) KU-2-8 after sorption (4:2); (**c**) KU-2-8 after sorption (3:3).

Figure 8 shows thermogravimetric (TG) and differential scanning calorimetric (DSC) curves for the following samples: (a) KU-2-8 (initial), (b) KU-2-8 from the system with the component ratio of 4:2 after sorption, and (c) KU-2-8 from the system with the ratio of 3:3. When comparing the graphs, it is evident that the initial weight loss for all three samples begins in a similar temperature range of 65–75 °C. For the initial KU-2-8, the weight loss in the range of 65–140 °C is more pronounced, whereas for samples (b) and (c), this region is characterized by a more uniform weight decrease. The temperature range of 50–150 °C is probably associated with the removal of adsorbed moisture, which is typical for cation exchangers. Upon reaching a temperature of 300 °C, the weight loss was (a) ~78%, (b) ~80%, and (c) ~86%. According to differential scanning calorimetry data, endothermic processes are observed in all three cases. Minor deviations in the DSC curve are recorded in the range of 220–230 °C, but the process as a whole retains an endothermic nature. The general nature of the TGA and DSC curves for the initial KU-2-8 sample and the samples after sorption is similar. Nevertheless, differences in mass losses may indicate that the presence of metal ions in the polymer structure increases the residual mass of the substance at a temperature of 300 °C, probably due to the interaction of metal ions with the polymer matrix or their contribution to the formation of thermally stable products.

**Figure 9:**
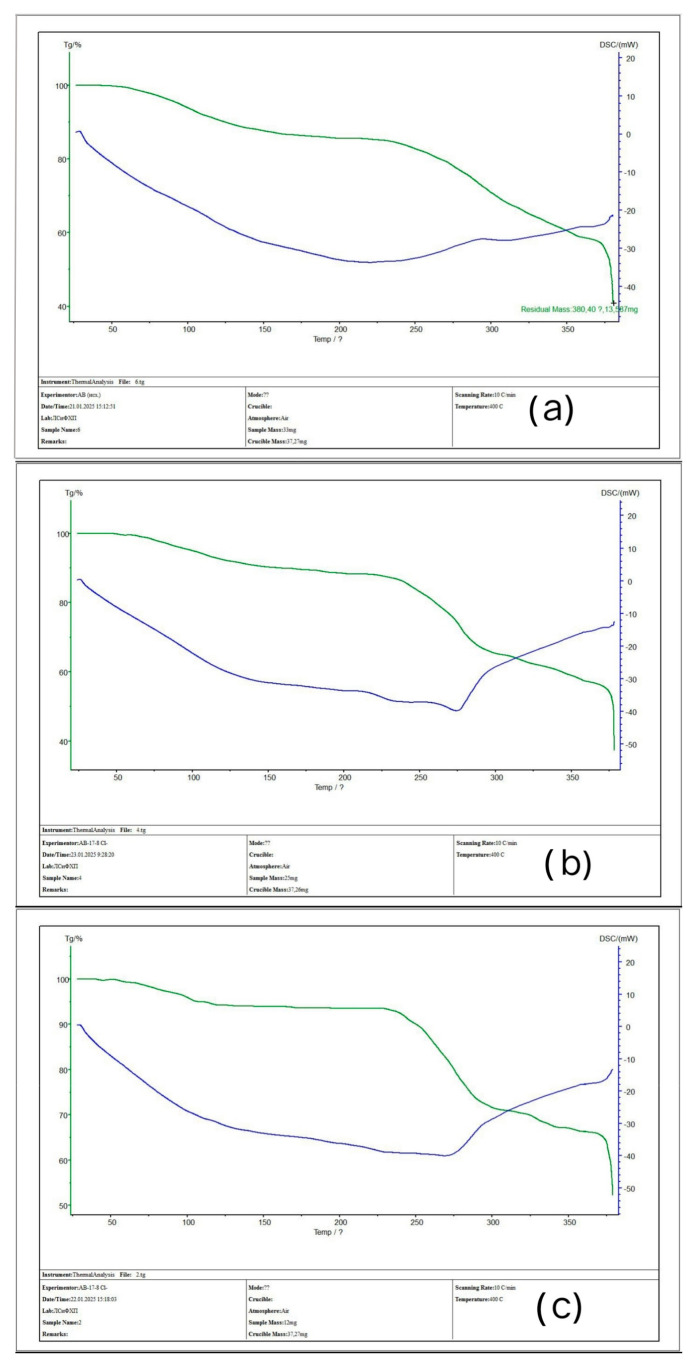
TG (green curve) and DSC (blue curve) graph: (**a**) initial AV-17-8 (not involved in sorption); (**b**) AV-17-8 after sorption (4:2); (**c**) AV-17-8 after sorption (3:3).

Figure 9 shows thermogravimetric (TG) and differential scanning calorimetric (DSC) curves for the following samples: (a) AV-17-8 (initial), (b) AV-17-8 from the system with the component ratio of 4:2 after sorption, and (c) AV-17-8 from the system with the ratio of 3:3. In all three cases, a smooth decrease in mass is observed in the temperature range of 0–220 °C. For the initial AV-17-8 (sample (a)), in the range of ~240–280 °C, there is a mass loss from ~87% to ~67%. For the AV-17-8 (4:2) sample, in the range of 230–310 °C, the mass loss is ~87% to ~65%, and the mass decrease line is more pronounced compared to the initial sample. For sample AV-17-8 (3:3), the process of weight loss is expressed even more clearly, and in this range, the weight changes from ~93% to ~71%. Comparison of three graphs in the temperature range of 50–150 °C allows for us to assume that adsorbed water is removed in this range. Analysis of DSC curves shows endothermic processes for all three samples. However, differences appear when comparing the initial anion exchanger and the samples after sorption. For the initial AV-17-8, the inflection on the DSC line is observed at a temperature of ~220 °C, while for samples (b) and (c), a similar inflection shifts to the region of ~275 °C. Despite the general nature of the TG and DSC curves, differences in the depth of weight loss are noticeable in the range of ~230–320 °C. It should also be noted that the rate of weight loss for samples (a) and (b) is higher compared to graph (c). Difficulties in interpreting the TG graphs of anion exchangers may be associated with different amounts of sorbed moisture, and also, probably, with the extremely low physical sorption of metal ions for samples from systems with a ratio of 4:2 and 3:3.

### 3.4. Study of Desorption of Target Metal Ions by Interpolymer Systems

The desorption results presented in Table 8 are interesting. First of all, considering the results of desorption from the cationite, it is easy to notice that in the dynamic mode, it was possible to obtain more of both ions than in the static mode. Moreover, the difference in the ratios of 4:2 and 3:3 is small in dynamic mode. Regardless of the sorption mode, neodymium was better desorbed from the polymer matrix compared to praseodymium. In static mode, a different picture is observed: the concentration of desorbed neodymium is almost twice as high as the concentration of praseodymium. This result may indicate a stronger retention of praseodymium in the cationite matrix. Presumably, this is due to conformational changes that occur as a result of the mutual influence of the cationite KU-2-8 and the anionite AV-17-8 during their joint activation. The interaction of KU-2-8 with praseodymium probably leads to the formation of more stable bonds, which may be due to either a specific (coordination) or non-specific (ionic) nature of the interactions. In the dynamic mode, constant mixing minimizes the effect of these energy differences, which leads to a more uniform desorption of both ions. However, even under these conditions, neodymium exhibits slightly better desorption (0.4 mg/L more) than praseodymium, which probably indicates the existence of non-random factors influencing the desorption process.

To get a clearer picture, it is necessary to calculate the degree of desorption in order to operate with percentage ratios. This value was calculated using the following formula:

(1) $$\omega =\frac{C_{desor.}}{C_{sorb.}}*100\%$$

where *ω* is the degree of desorption, *C_desor._* is the concentration of desorbed metal, *C_sorb_* is the concentration of absorbed metal. *C_sorb._* was calculated by subtracting the residual concentration from the initial ion concentration. The results for the degree of desorption for each ion are presented in the Table 9.

The degree of desorption shows that with active mixing both metals are almost equally extracted from the polymer matrix. In the static mode, a large difference appears between the desorption of the two metals. This leads to the conclusion that the choice of mode can affect the selectivity of both sorption and desorption. In the latter case, this is especially important, since the desired separation can be obtained at this stage (there is no point in selective sorption if it is impossible to obtain the target ion from the sorbent matrix at the next stage).

## 4. Conclusions

Interpolymer systems based on KU-2-8 and AV-17-8 are improved sorbents of neodymium and praseodymium ions. The choice of the sorption and desorption mode under the conditions of our experiment not only affects the amount of sorbed and desorbed ions, but also the selectivity of the entire process. In the static mode, high selectivity with respect to neodymium was observed. Thus, at a ratio of 4:2, neodymium was sorbed better than praseodymium by 24.16%, and in the 3:3 system by 39.83%. When desorption was carried out from the cation exchanger, a similar picture was also preserved: the concentration of neodymium was almost twice as high as the concentration of praseodymium. Sorption in dynamic mode in ratios of 4:2 and 3:3 does not lead to any selectivity, but a high degree of extraction of both ions is noted. Thus, the choice of conditions for the entire process greatly affects the depth and selectivity of sorption of Nd and Pr cations by interpolymer systems. Based on the above conclusions, such systems can be used for concentrating and separating REEs from each other using industrial solutions.

## Figures and Tables

**Table 1:** Some characteristics of the ion-exchange resins (KU-2-8 and AV-17-8) [24].

IERs	Fixed Groups	Polymer Matrix	True Density, g/cm^3^	Total Ion-Exchange Capacity, mmol cm^−3^
KU-2-8	-N^+^ (CH_3_)_3_	DVB8% + PS	1.13 [25]	1.12 ± 0.02 [26]
AV-17-8	-SO_3_^−^	DVB8% + PS	1.25 [25]	1.80 ± 0.01 [26]

**Table 2:** Residual concentration of neodymium and praseodymium after 48 h of sorption under different interaction conditions. (C_initial_ = 30 mg/L each).

PE Ratios and Sorption Mode	Nd (mg/L)	Pr (mg/L)
KU-2-8(Na):AV-17-8(Cl) 4:2 dynamic mode	1.3	0.192
KU-2-8(Na):AV-17-8(Cl) 3:3 dynamic mode	6.3	5.6
KU-2-8(Na):AV-17-8(Cl) 4:2 static mode	22.8	24.2
KU-2-8(Na):AV-17-8(Cl) 3:3 static mode	24.0	25.7

**Table 3:** Adsorption capacity for interpolymer systems in different modes.

PE Ratios and Sorption Mode	q (Nd) mg/g	q (Pr) mg/g
KU-2-8(Na):AV-17-8(Cl) 4:2 dynamic mode	72.65	75.46
KU-2-8(Na):AV-17-8(Cl) 3:3 dynamic mode	80.3	82.71
KU-2-8(Na):AV-17-8(Cl) 4:2 static mode	18.22	14.68
KU-2-8(Na):AV-17-8(Cl) 3:3 static mode	20.33	14.57

**Table 4:** pH values of interpolymer systems at different time intervals during sorption.

PE Ratios	pH (15 min)	pH (120 min)	pH (360 min)
KU-2-8(Na):AV-17-8(Cl) 4:2 dynamic mode	5.06	5.27	5.14
KU-2-8(Na):AV-17-8(Cl) 3:3 dynamic mode	4.97	5.19	4.98
KU-2-8(Na):AV-17-8(Cl) 4:2 static mode	5.04	4.94	5.09
KU-2-8(Na):AV-17-8(Cl) 3:3 static mode	5.05	5.16	5.30

**Table 5:** Extraction degree and extraction degree per 1 mole of praseodymium and neodymium ions by interpolymer systems.

PE Ratios and Sorption Mode	Nd Degree of Extraction %	Nd Degree of Extraction Per 1 mol in %	Pr Degree of Extraction %	Nd Degree of Extraction Per 1 mol in %
KU-2-8(Na):AV-17-8(Cl) 4:2 dynamic mode	95.67	23.91	99.36	24.81
KU-2-8(Na):AV-17-8(Cl) 3:3 dynamic mode	79	26.33	81.33	27.11
KU-2-8(Na):AV-17-8(Cl) 4:2 static mode	24.0	6	19.3	4.83
KU-2-8(Na):AV-17-8(Cl) 3:3 static mode	20.0	6.67	14.33	4.77

**Table 6:** Degree of polymer chain binding.

PE Ratios and Sorption Mode	θ (Nd) %	θ (Pr) %
KU-2-8(Na):AV-17-8(Cl) 4:2 dynamic mode	49.75	52.88
KU-2-8(Na):AV-17-8(Cl) 3:3 dynamic mode	54.78	57.72
KU-2-8(Na):AV-17-8(Cl) 4:2 static mode	12.48	10.29
KU-2-8(Na):AV-17-8(Cl) 3:3 static mode	13.87	10.17

**Table 7:** Effective dynamic sorption capacity.

PE Ratios and Sorption Mode	Q (Nd) mol/g	Q (Pr) mol/g
KU-2-8(Na):AV-17-8(Cl) 4:2 dynamic mode	0.0025	0.0026
KU-2-8(Na):AV-17-8(Cl) 3:3 dynamic mode	0.0027	0.0029
KU-2-8(Na):AV-17-8(Cl) 4:2 static mode	0.0006	0.0005
KU-2-8(Na):AV-17-8(Cl) 3:3 static mode	0.0007	0.0005

**Table 8:** Atomic emission analysis, desorption after 72 h (C initial = 30 mg/L each).

PE Ratios and Sorption Mode	Nd (mg/L)	Pr (mg/L)
KU-2-8(Na) 4:2 dynamic mode	15.8	15.4
KU-2-8(Na) 3:3 dynamic mode	14.6	14.2
AV-17-8(Cl) 4:2 dynamic mode	1.25	0.095
AV-17-8(Cl) 3:3 dynamic mode	1.52	0.33
KU-2-8(Na) 4:2 static mode	2.25	1.11
KU-2-8(Na) 3:3 static mode	2.24	1.09
AV-17-8(Cl) 4:2 static mode	1.32	0.17
AV-17-8(Cl) 3:3 static mode	1.33	0.18

**Table 9:** Degree of desorption of neodymium and praseodymium ions in different modes from the polymer matrix.

PE Ratios and Desorption Mode	(Nd) %	(Pr) %
KU-2-8(Na) 4:2 dynamic mode	55	51.66
KU-2-8(Na) 3:3 dynamic mode	61.6	58.19
KU-2-8(Na) static mode	31.25	19.13
KU-2-8(Na) static mode	37.33	25.34

## Data Availability

Data are contained within this article.
